# Wide-angle deep ultraviolet antireflective multilayers via discrete-to-continuous optimization

**DOI:** 10.1515/nanoph-2023-0102

**Published:** 2023-03-27

**Authors:** Jae-Hyun Kim, Dong In Kim, Sun Sook Lee, Ki-Seok An, Soonmin Yim, Eungkyu Lee, Sun-Kyung Kim

**Affiliations:** Department of Applied Physics, Kyung Hee University, Gyeonggi-do 17104, Yongin, Republic of Korea; Korea Research Institute of Chemical Technology (KRICT), Daejeon 34114, Republic of Korea; Department of Electronic Engineering, Kyung Hee University, Gyeonggi-do 17104, Yongin, Republic of Korea

**Keywords:** antireflective multilayer, calcium fluoride lens, deep ultraviolet spectrum, discrete binary optimization, factorization machine

## Abstract

To date, various optimization algorithms have been used to design non-intuitive photonic structures with unconventional optical performance. Good training datasets facilitate the optimization process, particularly when an objective function has a non-convex shape containing multiple local optima in a continuous parametric space. Herein, we developed a discrete-to-continuous optimization algorithm and confirmed its validity by designing and fabricating deep-ultraviolet antireflective MgF_2_/LaF_3_ multilayers. For discrete optimization, a multilayer was encoded into a binary vector with multiple bits; a 10 nm thick MgF_2_ or LaF_3_ layer was assigned a binary digit of 0 or 1, respectively. Using the binary-based training datasets, a factorization machine formulated a surrogate function, which discovered the ground binary vector representing a near-optimal figure of merit. Then, the figure of merit was refined through continuous optimization (e.g., using an interior-point method) of the ground binary vector. MgF_2_/LaF_3_ multilayers with a variety of bit levels were created to attain a minimum average angular (0°–45°) reflectance at 193 nm. A MgF_2_/LaF_3_ multilayer optimized at ten bits (i.e., a total thickness of approximately 100 nm) yielded an average reflectance of 0.2%, which agreed well with the experimental results. Moreover, an integrated ray-wave optics simulation predicted that a single CaF_2_ plano-convex lens coated with the optimized multilayer could exhibit a transmittance of 99.7%. The developed optimization approach will be widely applicable to any photonic structures that can represent a binary vector with multiple bits, such as microwave metasurfaces, in addition to being useful for producing ideal optical multilayers.

## 1 Introduction

Determining the ideal configuration of an optical multilayer that realizes a structured spectrum in specific ranges of wavelengths and angles is crucial yet challenging, as its structural parameters (e.g., number of materials, number of layers, and thickness of each layer) are unconstrained, allowing for the exploration of a vast parametric space [1–26]. The classical approach, which leverages constructive and destructive interference effects (e.g., multilayer Bragg mirrors and quarter-wavelength antireflection coatings [27]), can provide the optimal thickness of each layer when appropriate optical materials are accessible. However, more complex design strategies are required when the optical materials are substantially rare within a desired spectral range, such as in the deep ultraviolet (DUV) region (190–250 nm). Fluorides (e.g., CaF_2_, LaF_3_, AlF_3_, MgF_2_) are well known to be transparent in the DUV region [28–31].

In practice, an ArF excimer laser with a wavelength of 193 nm is used to photolithographically fabricate nanoscale semiconductor chips. Likewise, camera-based inspection equipment equipped with the same ArF excimer laser checks for compliance or non-compliance according to specific criteria during semiconductor processes [32]. For example, it detects any unintended defects in a wafer and coordinates their position. Multiple arrays of imaging systems with DUV-transparent CaF_2_ lenses are necessary for image-processing tasks in automated optical inspection. Therefore, incorporating an ideal antireflection coating into CaF_2_ lenses is critical for enhancing image quality. Particularly for high-numerical-aperture lenses, antireflection must be effective across a wide range of incident angles. In addition, the number of optical materials and total thickness should be minimized from an economic viability standpoint. In previous studies, Liu et al. fabricated a seven-layer MgF_2_/LaF_3_ antireflective coating (with a total thickness of 181 nm) that exhibited a reflectance of ∼0.5% at incident angles of 0°–45° at 193 nm [28]; Cangemi et al. achieved a reflectance of <0.2% at 193 nm for normal incidence by exploiting four distinct fluoride materials (GdF_3_/LaF_3_/AlF_3_/MgF_2_) in multilayers [33]; and using a HfO_2_/Al_2_O_3_/MgF_2_ trilayer, Kuschnereit et al. attained a reflectance of <0.5% at 193 nm, particularly for large incident angles of 72°–76° [34].

Various optimization strategies, such as needle optimization [13–16], memetic optimization [17–19], proximal policy optimization [20], and genetic algorithms [21–25], have been attempted in the search for multilayers that function in the ultraviolet, visible, and infrared spectral ranges. These inverse design methods have led to the creation of non-intuitive photonic structures, thus facilitating the development of spectrum-engineered applications such as radiative coolers [16, 18, 24, 25, 35], thermophotovoltaics [26], high-efficiency incandescent lighting [36], ultra-wideband absorbers [20], infrared-selective sensors [37], and infrared antireflective coatings [21]. A key process for optimization is to precisely track the gradient of an objective function, namely a figure-of-merit (FoM) or merit function. A FoM typically includes the quantitative difference in optical performance (e.g., average reflectance in this study) between the targeted and under-evaluated structures. Therefore, the minimum of a FoM reveals the ideal optical structure, which is achieved by tracking its gradient. However, if the parametric space of a FoM has a non-convex shape with multiple dispersed local minima (or maxima), the iterative optimization process becomes stalled close to one of the minima. Thus, the success of gradient-descent-based optimization is directly linked to the quality of the initial training data. Optimization using multiple starting points can resolve the issue, but determining how to identify good combinatorial datasets has yet to be clarified. As a result, establishing good training data is necessary for exploiting the capabilities of existing optimization methods.

Herein, we developed a two-step discrete-to-continuous optimization algorithm and applied it to the design of antireflective multilayers for DUV lenses. Discrete optimization (DO) employing a factorization machine (FM), which encodes a multilayer into a binary vector, can discover an initial state in the vicinity of a global minimum. Then, the predefined initial state is subjected to a continuous optimization (CO) process to minutely adjust the thickness of each layer and thus refine the FoM. When the DO-discovered structure is situated around an optimal state, the post-hoc convex optimization successfully identifies the best FoM. The devised two-step optimization discovered MgF_2_/LaF_3_ multilayers that assured a minimal average angular reflectance (0°–45°) at 193 nm while varying their total thicknesses in the range of 30–210 nm. The designer multilayer with a total thickness of 100 nm yielded an average reflectance of ∼0.2%, which was in good agreement with the experimental results. Moreover, an integrated ray-wave optics simulation predicted that a single CaF_2_ lens coated with the DUV antireflective multilayer could provide a transmittance of 99.7% at 193 nm without distorting the beam path or focal length.

## 2 Results and discussion

### 2.1 Discrete-to-continuous optimization

**Figure 1:**
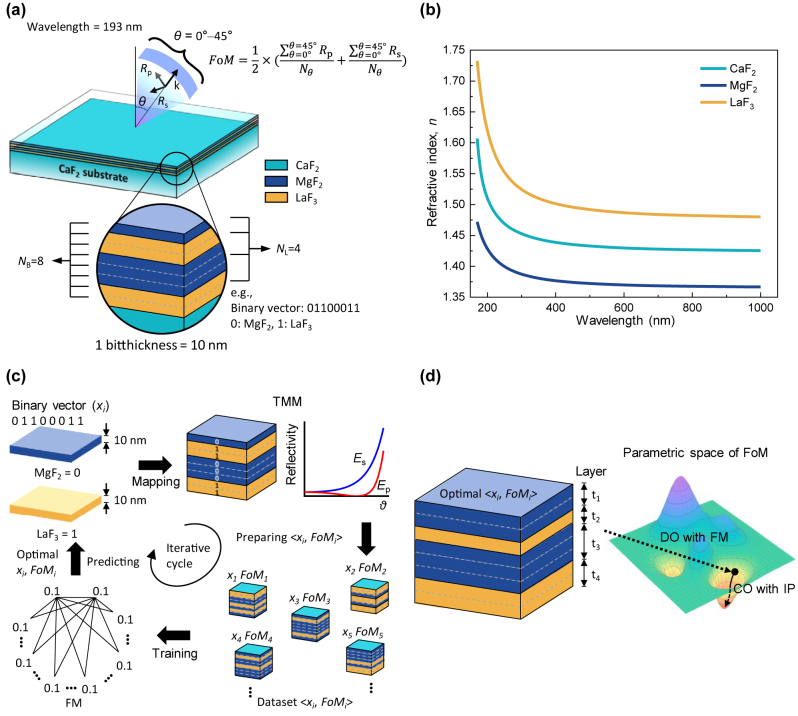
Principle of the developed two-step discrete-to-continuous optimization process. (a) Description of a FoM as an average reflectance across 0°–45° in the *p*- and *s*-polarizations, obtained at a single DUV wavelength (193 nm). A MgF_2_/LaF_3_ multilayer is composed of *N* bits (*N*_B_), where a 1 bit thickness is 10 nm, represented by a binary vector of 0 (MgF_2_) and 1 (LaF_3_). Note that *N*_L_ denotes the number of physical layers in a multilayer. (b) Measured refractive indices (*n*) of a CaF_2_ substrate, 60 nm thick MgF_2_ film, and 65 nm thick LaF_3_ film. (c) Schematics illustrating the iterative processes of a FM that discovers a DO-discovered ground binary vector for a DUV-antireflective MgF_2_/LaF_3_ multilayer: (i) encoding a binary vector into a multilayer (mapping), (ii) calculating the FoM of a given multilayer, (iii) updating the existing datasets <x_i_, FoM_i_> with a new dataset, and (iv) learning the optimum linear and quadratic coefficients of the surrogate model (training). (d) Schematics describing how to discover an optimized FoM via the developed two-step (DO with FM + CO with IP) optimization process.

In our design, the DUV antireflective multilayers consist of MgF_2_/LaF_3_ binary fluorides on a CaF_2_ substrate (Figure 1(a)). The material and thickness of each physical layer should be optimized in a multilayer with the total thickness constraint. We tackled this problem using a combined approach of DO and CO (Methods in the Supplementary Information). For the DO process, a given multilayer was defined as a binary vector with multiple bits (*N*_B_) by assigning a 10 nm thick MgF_2_ (or LaF_3_) layer a binary digit of 0 (or 1). For example, a binary vector of {0 1 1 0 0 0 1 1} with eight bits (*N*_B_ = 8) corresponds to a multilayer composed of 10 nm MgF_2_/20 nm LaF_3_/30 nm MgF_2_/20 nm LaF_3_ (see the low part of Figure 1(a)). It should be noted that the number of physical layers (*N*_L_) can differ from *N*_B_; for the multilayer with *N*_B_ = 8, *N*_L_ = 4. A binary vector (*x*) representing a multilayer has a FoM defined as follows:

(1) $$FoM=\frac{1}{2}\times \left[\frac{\int_{\theta =0}^{\theta =\theta_{f}}R_{p}\left(\theta \right)d\theta }{\int_{\theta =0}^{\theta =\theta_{f}}d\theta }+\frac{\int_{\theta =0}^{\theta =\theta_{f}}R_{s}\left(\theta \right)d\theta }{\int_{\theta =0}^{\theta =\theta_{f}}d\theta }\right]$$

where *R*_s_(*θ*) or *R*_p_(*θ*) is a reflectance at 193 nm averaged over a specific range of incident angles (0°–45° for this study) in the s- or p-polarization direction. To evaluate *R*_s_(*θ*) and *R*_p_(*θ*), we performed transfer matrix method (TMM) simulations using the measured refractive indices of the MgF_2_, LaF_3_, and CaF_2_ materials shown in Figure 1(b) (Methods in the Supplementary Information). In Equation (1), the lower the FoM, the better the antireflection performance; a perfect antireflective multilayer has *R*_s_ = *R*_p_ = 0, thus yielding a FoM of 0. Therefore, the DO process aims to discover the ground binary vector x* that has the lowest FoM at a given *N*_B_, as follows:

(2) $$x^{*}=\underset{x\in \left[0,1\right]}{arg\;min}FoM$$

However, it is computationally expensive to investigate all possible states and their associated forms using the TMM because the total number of states is proportional to 2 to the power of *N*_B_. To avoid this time-consuming process, we constructed a quadratic unconstrained binary optimization (QUBO) model and used it to search for the ground binary vector [38] (Methods in the Supplementary Information). For the QUBO model, a set of {*X*, *f*}, referred to as the training dataset, was prepared, where *X* = {*x*_1_, *x*_2_, …, *x*_*m*_} and *f* = {FoM_1_, FoM_2_, …, FoM_*m*_}. Then, the model was formulated with the training dataset using a factorization machine (FM), as follows:

$$f^{\prime }=w_{0}+w^{T}X+1^{T}\left[\left(VX\right)◦\;\left(VX\right)-\left(V◦V\right)\left(X◦X\right)\right]$$

(3) $${w_{0}}^{*},w^{*},V^{*}=arg\;min\;L\left(f,f^{\prime }\right)$$

where $w_{0}\in R^{1}$ , $w\in R^{N_{\text{bit}}}$ , $V\in R^{m\times N_{\text{bit}}}$ , **1** ∈ [1]^*m*^, *L*(***f***, ***f*′**) is the loss function with L2 regularization, and m is a positive integer. A stochastic gradient method was used to fit the hyperparameters (i.e., *w*_0_, ***w***, V) of the model. Then, a surrogate function (***s*** (*X*)) was formulated as follows:

(4) $$\begin{array}{ll} s\left(X\right) & ={w_{0}}^{*}+w^{*T}X+1^{T}\left[\left(V^{*}X\right)◦\;\left(V^{*}X\right)\right.\\ & \;\left.-\left(V^{*}◦V^{*}\right)\left(X◦X\right)\right] \end{array}$$

Since the surrogate function mimicked a FoM, the problem of searching for the ground binary vector (x*) yielding the lowest (i.e., optimal) FoM in Equation (2) was solved by searching for x*, yielding the lowest ***s*** value:

(5) $$x^{*}=\underset{x\in \left[0,1\right]}{arg\;min}\;s\left(x^{*}\right)$$

This surrogate function deals with the set of ***x*** (i.e., tensor *X* $\in {\left[0,1\right]}^{n\times N_{\text{B}}}$ ) per calculation, which makes the optimization algorithm fast and efficient.

After the surrogate function identified the ground binary vector, the TMM evaluated the corresponding multilayer to validate whether the binary vector yielded the lowest FoM in the training datasets and whether ***s*** was identical to the actual FoM. The training dataset was updated by adding the binary vector and its associated FoM. Then, a more accurate surrogate function was formulated with the updated training dataset, and discovering the ground binary vector of the FoM became more probable. The whole cycle of the iterative optimization process is schematically depicted in Figure 1(c). The iterative cycle continued until a FoM converged to a single value. In this way, the DO process with a FM discovered the ground binary vector of a FoM at a given *N*_B_. Sequentially, the CO process with an interior-point (IP) method, which is known as a non-linear convex optimization, was initiated using the DO-discovered binary vector (i.e., the ground binary vector of a FoM). The post-hoc CO process led to a modest refinement of the thickness of each layer to within a 1 bit thickness limit (i.e., 10 nm) (Figure 1(d)). For example, for *N*_B_ = 14 (*N*_L_ = 6), the first (MgF_2_) and fifth (MgF_2_) layers from the bottom had thicknesses of 10 and 40 nm, respectively, after the first-round DO process was complete. Subsequently, after the post-hoc CO process, their thicknesses changed to 19.9 and 32.4 nm in sequential order.

### 2.2 Design of DUV antireflective multilayers

**Figure 2:**
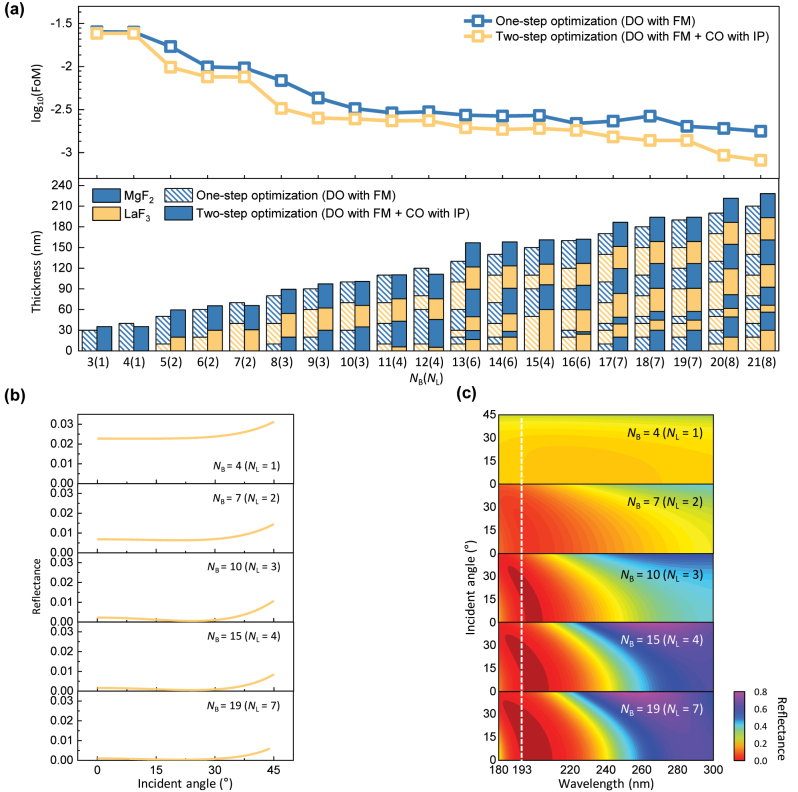
Simulated results of DUV antireflective multilayers optimized at various bit levels. (a) Simulated FoM values (top) and structural information (i.e., the material and thickness of each layer) (bottom) of DUV antireflective multilayers as a function of *N*_B_ (*N*_L_), obtained through one- (DO with FM) and two-step (DO with FM + CO with IP) optimizations. (b) Simulated angular (0°–45°) reflectance spectra of DUV antireflective multilayers optimized at various *N*_B_ (*N*_L_) values. The incident wavelength is 193 nm. (c) Simulated angle-resolved reflectance spectra of DUV antireflective multilayers optimized at various *N*_B_ (*N*_L_) values.

We designed DUV antireflective multilayers via a two-step optimization (DO with FM + CO with IP) at various bits of binary vectors. Figure 2(a) shows the optimized FoM values (top panel) and their corresponding multilayer configurations (i.e., the material and thicknesses of each layer) (bottom panel) as a function of *N*_B_, obtained after one- (DO with FM) and two-step (DO with FM + CO with IP) optimization processes. For both cases, the FoM steadily decreased with increasing *N*_B_; for example, the one-step (or two-step) optimization discovered FoMs of 0.025 (or 0.024) and 0.003 (or 0.002) at *N*_B_ = 3 and 10, respectively. The FoM was almost clamped at 10 ≤ *N*_B_ ≤ 19, slightly reduced to 0.0009 at *N*_B_ = 20, and finally reached the lowest value of 0.0008 at *N*_B_ = 21 (corresponding to *N*_L_ = 8). The post-hoc process (i.e., CO with IP) slightly improved the antireflection performance. Between one-step (DO with FM) and two-step (DO with FM + CO with IP) optimizations, the difference in FoM is smaller than 0.001 at 3 ≤ *N*_B_ ≤ 21. At certain *N*_B_ values (e.g., *N*_B_ = 10, 11, and 16), both optimizations yielded very similar multilayer configurations and their resultant FoMs. These findings suggest that the DO with FM can find the ground binary vector of the FoM at a given *N*_B_, which is close to the performance of an ideal multilayer.

We obtained the reflectance of multilayers optimized at *N*_B_ = 4, 7, 10, 15, and 19 as a function of the incident angle (0–45°) (Figure 2(b)). Notably, the reflectance was minimized near 25° irrespective of *N*_B_ because the FoM represented an average reflectance across 0°–45°. At *N*_B_ = 19 (corresponding to *N*_L_ = 7), the optimized multilayer exhibited a reflectance of <0.13% for the considered incident angles. The seven-layer film of alternating binary fluorides had a thickness of ∼190 nm. Nevertheless, its wide-angle antireflection performance surpassed those reported in previous studies [28, 33, 34]. The overall shape and amplitude of the angular reflectance spectrum marginally changed when *N*_L_ = 3, 4, and 7, except for the extreme data points at the incident angles near 0°and 45°. Also, the multilayers with *N*_L_ = 3, 4, and 7 retained their superior wide-angle antireflection performance in the wavelength range of 180–200 nm (Figure 2(c)), which is indicative of broadband antireflection and fabrication tolerance.

A phase shift occurs negligibly as light propagates over a distance of less than one-tenth of the effective wavelength. Therefore, a 1 bit thickness was chosen as 10 nm. To support this, we obtained FoM values through the DO process with 1 bit thicknesses of 10 or 20 nm (Supporting Information Figure S1). For MgF_2_/LaF_3_ multilayers of the equal total thickness, the 10 nm condition resulted in significantly lower FoM values, indicating its suitability as an initial state for the post-hoc CO process. We note that adopting a 1 bit thickness of less than 10 nm does not lead to a superior initial state and incurs substantial computational costs. Nonetheless, CO successfully hones the thickness of each layer within 10 nm.

Fluorides, such as CaF_2_, LaF_3_, AlF_3_, and MgF_2_, exhibit transparency in the DUV region. Even if they exhibit some degree of absorption, the reflectance of multilayers is only marginally affected due to the weak optical resonance of antireflection. To verify this, we acquired angular transmittance spectra of MgF_2_/LaF_3_ multilayers optimized at *N*_B_ = 10 with extinction coefficients (*k*) of both fluorides set to 0 (i.e., transparent) or 0.001 (i.e., weakly absorptive). The two spectra displayed good agreement, with the average angular (0°–45°) transmittance values being 99.75% and 99.05% for *k* values of 0 and 0.001, respectively (Supporting Information Figure S2).

### 2.3 Experimental verification of designer multilayers

**Figure 3:**
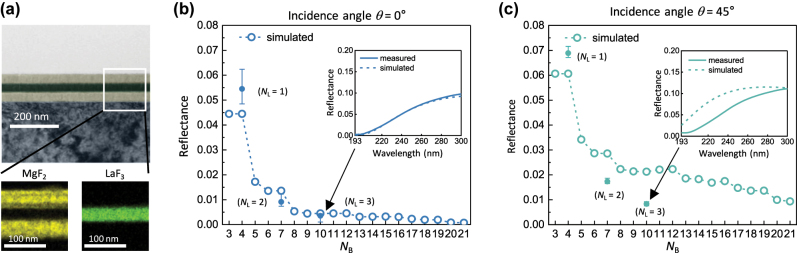
Analysis of the fabricated DUV antireflective multilayers. (a) Cross-sectional TEM and EDX images of fabricated DUV-antireflective tri-layer films. The blue, yellow, and green (false-colored) areas indicate CaF_2_, MgF_2_, and LaF_3_, respectively. (b and c) Calculated and measured reflectance values of DUV-antireflective multilayers when the incidence angle is 0° (b) and 45° (c). In each inset, the calculated and measured reflectance spectra of the same structures are shown.

We chose three DUV antireflective multilayers optimized at *N*_B_ = 4, 7, and 10 (corresponding to *N*_L_ = 1, 2, and 3) to experimentally validate the results derived from the simulation design. The designed multilayers were coated on the front and back sides of a 2 mm thick CaF_2_ substrate using a standard thermal evaporator (Methods in the Supplementary Information). Double-sided antireflection coatings are essential to maximize the transmittance of lenses. The cross-sectional transmission electron microscopy (TEM) images with energy-dispersive X-ray (EDX) elemental mapping data revealed that each layer had a controlled thickness that consisted of homogenous atomic elements, and its interface was sharply discernible (Figure 3(a)). Optical simulations showed that the thickness of the topmost layer was the most critical to the antireflection performance (Supporting Information Figure S3).

We obtained the reflectance values of the fabricated samples at two incident angles of 7° and 45° using a UV–visible spectrophotometer (Methods in the Supplementary Information). For both incident angles, the reflectance rapidly dropped with increasing *N*_L_, which is consistent with the simulated data (Figure 3(b) and (c)). For the near-normal incidence, the sample with *N*_L_ = 3 displayed the best antireflection performance, with a reflectance of ∼0.35%. Its measured reflectance spectrum (190–300 nm) is in good agreement with the simulated data (inset, Figure 3(b)). To verify the impact of topmost MgF_2_ layer discussed in Supporting Information Figure S3, we fabricated three DUV-antireflective multilayers with *N*_L_ = 3, in which only the thickness of topmost layer was detuned from an optimum value (Supporting Information Figure S4). At an incident angle of 45°, the measured reflectance values were even lower than the simulated data at *N*_L_ = 2 and 3, but their qualitative behavior with increasing *N*_L_ was similar (Figure 3(c)). We speculate that the discrepancy in absolute reflectance results from the dependence of the refractive index on film thickness [39, 40]; in Figure 1(b), the refractive indices of the MgF_2_ and LaF_3_ materials with thicknesses of 60 nm and 65 nm, respectively, were determined. Specifically, at 45° incidence, the magnitude of the propagation vector becomes relatively small, thereby causing any potential mismatches in interference conditions and leading to a more pronounced discrepancy.

### 2.4 Simulation of antireflective-multilayer-coated CaF_2_ lenses

**Figure 4:**
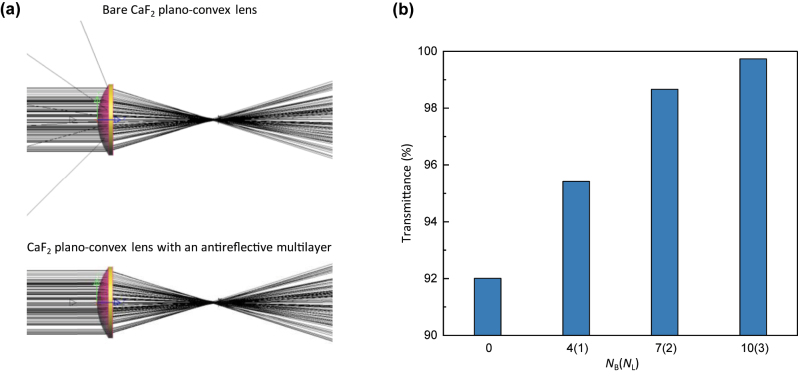
Simulated results of a CaF_2_ lens coated with DUV antireflective multilayers. (a) Simulated ray trajectories when light passes through a single CaF_2_ plano-convex lens (radius of curvature of 0.024 mm, diameter of 50 mm, and focal length of 97.74 mm) with or without a DUV antireflective multilayer (*N*_L_ = 3) using the developed integrated ray-optics simulation. (b) Simulated transmittance of a CaF_2_ lens when coated with DUV antireflective multilayers optimized at various *N*_L_ values. Note that *N*_L_ = 0 indicates a bare CaF_2_ lens.

The optimized antireflection coatings have the potential to greatly improve the transmittance of DUV CaF_2_ lenses. To verify this, we conducted integrated ray-wave optics simulations on a CaF_2_ plano-convex lens with and without an optimized MgF_2_/LaF_3_ multilayer coating [41] (Methods in the Supplementary Information). To implement the integrated ray-wave optics simulation, the angular reflectance information of an optimized multilayer was transferred to the surface of a CaF_2_ lens with a specific shape, curvature, and diameter. Then, a ray-tracing method was used to simulate the trajectory and transmitted intensity of incident rays that passed through the surface-engineered CaF_2_ lens. The ray-tracing simulations indicated that the optimized multilayer coating did not alter the beam trajectory through the lens; the focal length remained unchanged (Figure 4(a)). More importantly, the multilayer coatings remarkably enhanced the transmittance (Figure 4(b)). The transmittance of the multilayer-coated CaF_2_ lens steadily improved with increasing *N*_L_ and reached 99.7% at *N*_L_ = 3.

## 3 Conclusions

In conclusion, we developed a two-step optimization algorithm and applied it to design DUV antireflective multilayers. The DO with FM in the first optimization round discovered a near-perfect structure, which was a good initial state for the CO with IP in the second optimization round. The sequential discrete-to-continuous (i.e., coarse-to-fine) design strategy quickly created an optimal antireflective multilayer at a given total thickness (i.e., *N*_B_). The designed MgF_2_/LaF_3_ multilayer with *N*_B_ = 21 (or *N*_L_ = 8) attained an average angular (0°–45°) reflectance of <0.1% at 193 nm. Measurements on the fabricated MgF_2_/LaF_3_ multilayers with various *N*_B_ levels confirmed the validity of the two-step optimization algorithm. The antireflection performance (e.g., transmittance and bandwidth) of the multilayers could be further improved if they were designed using more DUV-transparent materials. For example, a multilayer with four candidate materials would be encoded with the combination of two binary vector labels: (00), (01), (10), and (11) [12]. Such a scheme readily converts quaternary or even octal optimization problems into binary ones based on the QUBO model. Moreover, the devised optimization algorithm is not only effective for providing optimal optical multilayers but is also broadly applicable to any photonic structures that can represent a binary vector with multiple bits (e.g., microwave metasurfaces [42, 43]). However, for the optimization of two- and three-dimensional subwavelength structures, a larger number of bits is required. In such instances, quantum annealing may be a useful approach for implementing the DO with FM for the first-round discrete optimization [12]. We believe that the existing convex and non-convex optimization methods will benefit from the DO with FM method by establishing good starting points.

## Supplementary Material

Supplementary Material Details
