# Supplementary material for: Wide-angle deep ultraviolet antireflective multilayers via discrete-to-continuous optimization
Source: Nanophotonics. 2023 Mar 27;12(10):1913–21. doi: 10.1515/nanoph-2023-0102 (PMC11501509; doi:10.1515/nanoph-2023-0102)
Supplement: Supplementary file 1 — Supplementary Material Details [file j_nanoph-2023-0102_suppl_001.docx]

Supplementary Materials

Jae-Hyun Kim^1^, Dong In Kim^2^, Sun Sook Lee^2^, Ki-Seok An^2^, Soonmin Yim^2^*, Eungkyu Lee^3^*, Sun-Kyung Kim^1^*

**Wide-angle deep ultraviolet antireflective multilayers via discrete-to-continuous optimization**

^1^Department of Applied Physics, Kyung Hee University, Gyeonggi-do 17104, Republic of Korea

^2^Korea research institute of chemical technology (KRICT), Daejeon 34114, Republic of Korea

^3^Department of Electronic Engineering, Kyung Hee University, Gyeonggi-do 17104, Republic of Korea

*S.-K.K. (sunkim@khu.ac.kr)

*E.L. (eleest@khu.ac.kr)

*S.Y. (s.yim@krict.re.kr)

**1 Optical characterization**

To obtain the reflectance spectra at near- (7°) and off-normal (45°) incidence, a spectrophotometer (Cary 5000, Varian) was used with a specular reflectance accessory (VW Absolute Specular Reflectance Accessory, Varian). The incident light source and detector could not be placed in the same location owing to geometrical constraints. Therefore, 7° was chosen to emulate normal incidence. For the 45°-reflectance measurement, the same spectrometer was used with a different accessory (UV–Vis–NIR Angle Specular Reflectance Accessory, Varian). A 200 nm thick sputtered Al film (SME-200J, ULVAC) was used as a reference sample for the reflectance measurement. The manufacturer of the DUV mirror provides a tabulated reflectance spectrum at normal incidence. The optical constants of the reference Al film were obtained over the UV and visible regions (180–800 nm) using a spectroscopic ellipsometer (Elli-SE, Ellipso Technology). The reflectance spectrum of the reference Al film was determined using the measured optical constants. Finally, the reflectance spectra of the fabricated DUV antireflective samples were obtained by normalizing their measured reflected spectra with that of the reference sample.

**2 Fabrication of DUV antireflective coatings**

Single and multilayer antireflection coatings were performed on commercialized CaF_2_ substrates (2 mm thickness, EKSMA optics). In addition, Si substrates (DASOM RMS) were used as a reference for ex-situ monitoring. Prior to film deposition, both substrates were cleaned via sonication with acetone, isopropanol, and distilled water for 30 min each. MgF_2_ (99.99%, TASCO) and LaF_3_ (99.95%, TASCO) films were deposited using a thermal evaporator with two boats, and the chamber pressure was 10–6 Torr during the film deposition. MgF_2_ sources (1–4 mmcs, pellets) were used as purchased, while the LaF_3_ granules were ground and used in powdered form to facilitate a reliable deposition rate. The distance between the source and substrate was approximately 30 cm, and the substrates were not intentionally heated during the process. MgF_2_ and LaF_3_ thin-films were sequentially deposited with a constant deposition rate of 0.3–0.5 Å/sec; the deposition rate and accumulated thickness were monitored through a quartz crystal monitor. The obtained film thickness and refractive indices were confirmed with the reference Si substrates using a spectroscopy ellipsometer (UVISEL Plus, HORIBA).

**3 Integrated ray-wave optics simulations**

We conducted ray-tracing simulations using commercial software (LightTools, Synopsys Inc.). The wave-optics simulations were performed using a TMM built in-house. We imposed the results (i.e., transmittance and reflectance angular spectra (0°–45°) at 193 nm) from the wave-optics simulations into the surface of a CaF_2_ lens. The diameter, focal length, and curvature of a plano-convex CaF_2_ lens in the ray-tracing simulation model were 50 mm, 97.74 mm, and 0.024 mm, respectively. A number (100,000) of parallel rays were incident on the lens to analyze the beam trajectory and transmittance.

**4 Two-step discrete-to-continuous optimization**

An open-source factorization machine package (xLearn) was used for the discrete optimization (DO) process. The training datasets were prepared with the transfer matrix method (TMM) customized in MATLAB, where they consisted of 25 randomly generated structures (binary vectors) and figure-of-merits (FoMs). The FM learned the hyperparameters (i.e.,$w_{0}$, $\boldsymbol{w}$, $V$) from the training datasets by minimizing $L(\boldsymbol{f}, \boldsymbol{f}')$ using a stochastic gradient method with a learning rate of 0.001, a regularization parameter of 0.001, and epochs of 1000. For $V$, the length of latent vector m was fixed to 8. For continuous optimization (CO), an interior point method in the MATLAB optimization toolbox (fmincon) was used. To efficiently perform the CO process, the thickness of each layer was normalized to 500 nm, and the interior point method had the minimum and maximum bounds of a normalized thickness of 0.01 and 1, respectively. All computational processes were performed on a workstation with 512 GB and an AMD RyzenTM ThreadripperTM PRO 5995WX, 64-Core.

**Figure S1**: Simulated results obtained with one-step (DO with FM) optimization based on 1-bit thicknesses of 10 or 20 nm. FoM values (top) and structural information (i.e., the material and thickness of each layer) (bottom) as a function of total thickness.

**Figure S2**: Simulated angular (0°–45°) transmittance spectra of DUV antireflective multilayers optimized at different *N*_B_ (*N*_L_) values when transparent (*k* = 0) or weakly absorptive (*k* = 0.001) MgF_2_ and LaF_3_ materials are used.

**Figure S3:** Fabrication tolerance of DUV antireflective multilayers. (a) Simulated FOM values of DUV antireflective multilayers with *N*_L_ = 3 only when the thickness of a specific layer is detuned from an optimum value. (b) Cross-sectional TEM and EDX (false-colored) images of fabricated DUV antireflective multilayers with *N*_L_ = 3 with different thicknesses of the topmost MgF_2_ layer. The yellow and green areas indicate MgF_2_ and LaF_3_, respectively.

**Figure S4:** Experimental verification of the designed antireflective multilayers. Measured and simulated reflectance values of DUV antireflective multilayers with *N*_L_ = 3 as a function of the thickness of the topmost MgF_2_ layer. Note that the other MgF_2_ and LaF_3_ layers have fixed optimal thicknesses.
